# Luminal STAT5 mediates H2AX promoter activity in distinct population of basal mammary epithelial cells

**DOI:** 10.18632/oncotarget.9718

**Published:** 2016-05-30

**Authors:** Moshe Reichenstein, Gat Rauner, Shenhav Kfir, Tatiana Kisliouk, Itamar Barash

**Affiliations:** ^1^ Institute of Animal Science, ARO, The Volcani Center, Bet-Dagan, Israel; ^2^ The Robert H. Smith Faculty of Agriculture, Food and Environment, The Hebrew University of Jerusalem, Rehovot, Israel

**Keywords:** STAT5, H2AX, mammary gland, methylation, paracrine

## Abstract

Deregulated STAT5 activity in the mammary gland causes parity-dependent tumorigenesis. Epithelial cell cultures transfected with constitutively active STAT5 express higher levels of the histone H2AX than their non-transfected counterparts. Higher H2AX expression may be involved in tumorigenesis. Here, we aimed to link high STAT5 activity to H2AX–GFP expression by looking for distinct types of mammary cells that express these proteins. *In vitro* and in transgenic mice, only 0.2 and 0.02%, respectively, of the cells expressed the H2AX–GFP hybrid gene. Its expression correlated with that of the endogenous H2AX gene, suggesting that detectable H2AX–GFP expression marks high levels of H2AX transcript. Methylation of the H2AX promoter characterized non-GFP-expressing H2AX–GFP cells and was inversely correlated with promoter activity. Administration of 5-azacytidine increased H2AX promoter activity in an activated STAT5-dependent manner. In transgenic mice, H2AX–GFP expression peaked at pregnancy. The number of H2AX–GFP-expressing cells and GFP expression decreased in a Stat5a-null background and increased in mice expressing the hyperactivated STAT5. Importantly, H2AX–GFP activity was allocated to basal mammary cells lacking stem-cell properties, whereas STAT5 hyperactivity was detected in the adjacent luminal cells. Knockdown of RANKL by siRNA suggested its involvement in signaling between the two layers. These results suggest paracrine activation of H2AX via promoter demethylation in specific populations of basal mammary cells that is induced by a signal from neighboring luminal cells with hyper STAT5 activity. This pathway provides an alternative route for the luminally confined STAT5 to affect basal mammary cell activity.

## INTRODUCTION

Two main epithelial cell populations are orchestrated during mammary gland development and activity: (i) the apically oriented luminal epithelial cells that line the duct or alveolar lumen, and are involved in the synthesis of milk components and milk secretion; (ii) the basally oriented myoepithelial cells that contact the basement membrane. Upon hormonal stimulation, these latter cells contract and direct the secreted milk toward the nipple [1, 2]. The basal compartment of the epithelium also encompasses a mammary repopulating unit (MRU)—a small subpopulation of cells that can generate an entire functional gland upon transplantation into the de-epithelialized (cleared) mammary fat pad of a host mouse. Collectively, the mammary epithelial cells are organized into a series of branching ducts that terminate in secretory alveoli during lactation [3, 4]. Considerable heterogeneity has been found within the mammary tissue, and cell subpopulations can be defined within a given cell lineage, including the stem-cell fraction [5]. For example, mammary ductal expansion and branching depend on systemic estrogen and progesterone. However, only ~10–15% of luminal epithelial cells in the normal breast express immunodetectable estrogen receptor (ER) α [6], and about 7% express progesterone receptor (PR) [7].

Regulation of luminal cell differentiation has been extensively studied in the mammary gland [3], whereas the characteristics of the basal lineage are only now emerging [8, 9]. A major regulator of luminal cell differentiation and activity is signal transducer and activator of transcription 5 (STAT5). Stat5a mediates the generation of alveolar progenitors from mammary stem cells [10] and is therefore mandatory for mammary gland development during pregnancy [11]. Furthermore, it controls cell differentiation and lactogenesis, as well as epithelial cell survival [12, 13]. Prolactin (PRL) is the main inducer of Stat5a transcriptional activity in the mammary gland and glucocorticoid receptors enhance its transactivation through protein–protein interactions (reviewed by [14, 15]). In the absence of Stat5a, increased expression of the Stat5b variant occurs after the first pregnancy and rescues the lactation defect [16]. However, unlike Stat5a, deletion of Stat5b has no effect on mammary phenotype [17, 18].

Deregulated Stat5 activity in the hematopoietic system is associated with the development of Hodgkin's lymphoma and Burkett's lymphoma in humans [19] and, via the kinase BCR–ABL, with myeloid leukemia [20]. In the mammary gland, studies in transgenic animal models have indicated that deregulated STAT5 levels and activity represent a genuine risk factor for breast cancer [21]. Deregulated STAT5 activity resulted in parity-dependent latent development of tumors in transgenic mice [22–24] and in reconstituted glands formed by transplantation of lenti-transfected epithelial cells expressing the constitutively active variant of Stat5 (ovine Stat5 which is homologous to the murine Stat5a and termed here STAT5 [12]). Since expression and activity of the STAT5 transgene and the endogenous murine Stat5a are indistinguishable, the term STAT5 is used hereafter for both transgenic and transfected cells. Two basic perceptions concerning STAT5 involvement in tumorigenesis have been revealed: (i) pregnancy is probably the risk period for the initiation of STAT5′s oncogenic activity [25]; (ii) the onset of STAT5-dependent tumors engulfs a few individual cells with hyperactive STAT5 [23]. Morphological examinations, complemented by gene array and bioinformatics analyses, have suggested that the ability of STAT5 variants to induce tumorigenesis involves activation of the DNA damage response (DDR) pathway [25, 26]. The involvement of STAT5-mediated, PRL-induced oxidative stress in the induction of H2AX, CHK2 and BRCA1 genes during a pregnancy-like state has been shown *in vitro* [25]. Interestingly, a distinct cell population has been identified in the breast that evades the mechanisms which evolved to prevent the propagation of cells with oxidatively damaged DNA [27].

H2AX is a member of the histone 2A (H2A) family, one of the five families of histone proteins involved in the nucleosomal organization of chromatin [28]. H2AX is encoded by an alternatively processed transcript that yields two mRNA species—a 0.6-kb stem–loop transcript that is indistinguishable from those of replication-linked histones, and a 1.6-kb read-through polyadenylated transcript which has been detected in all examined cell lines. The human H2AX gene promoter has been partially characterized [28], but less information is available regarding its murine counterpart. The best known function of H2AX is associated with the DDR system, involving its induction by DNA double-strand breaks. H2AX is phosphorylated on S139 in the C-terminal of the H2AX tail, yielding a specific modified form known as γH2AX that promotes the recruitment of DNA-repair proteins to the site of the double-strand break [29, 30]. In mammary epithelial cells, oxidative stress induced by forced-activated STAT5 under pregnancy-like conditions also caused elevated H2AX expression [25]. Apparently, expression of H2AX has a double-edged regulatory role in tumorigenesis. On the one hand, elevated H2AX levels help prevent aberrant repair of both programmed and general DNA breakage and thus function as a dose-dependent suppressor of genomic instability and tumors in mice [31, 32]. On the other, p53-mediated H2AX downregulation is required to maintain normal embryonic fibroblast cell quiescence. Transfection of an H2AX expression vector that increased H2AX expression in these cells resulted in an accelerated rate of immortality [33]. In addition, H2A has been recently associated with resistance to anthracycline treatment for breast cancer [34]. These data emphasize the importance of highly controlled levels of H2AX expression for cell homeostasis.

The aim of this study was to identify individual cell populations that are prone to STAT5-dependent tumorigenesis by focusing on lactogenic hormone-responsive, STAT5-sensitized cells with elevated H2AX promoter activity. These cells represent a candidate core for cell transformation. Here, we identified a rare mammary basal cell subpopulation with H2AX promoter activity that is enhanced in response to paracrine signal from neighboring luminal cells. This signal, which may involve RANKL secretion, seems to be exclusively generated by lactogenic hormone-responsive luminal cells with hyper STAT5 activity and to cause hypomethylation of the H2AX proximal promoter in their neighboring basal counterparts.

## RESULTS

### Lactogenic hormone supplementation increases the number of CID-9 cells expressing H2AX fused to green fluorescent protein (GFP) in a STAT5-dependent manner. H2AX promoter activity is correlated with expression of the endogenous gene

**Figure 1:**
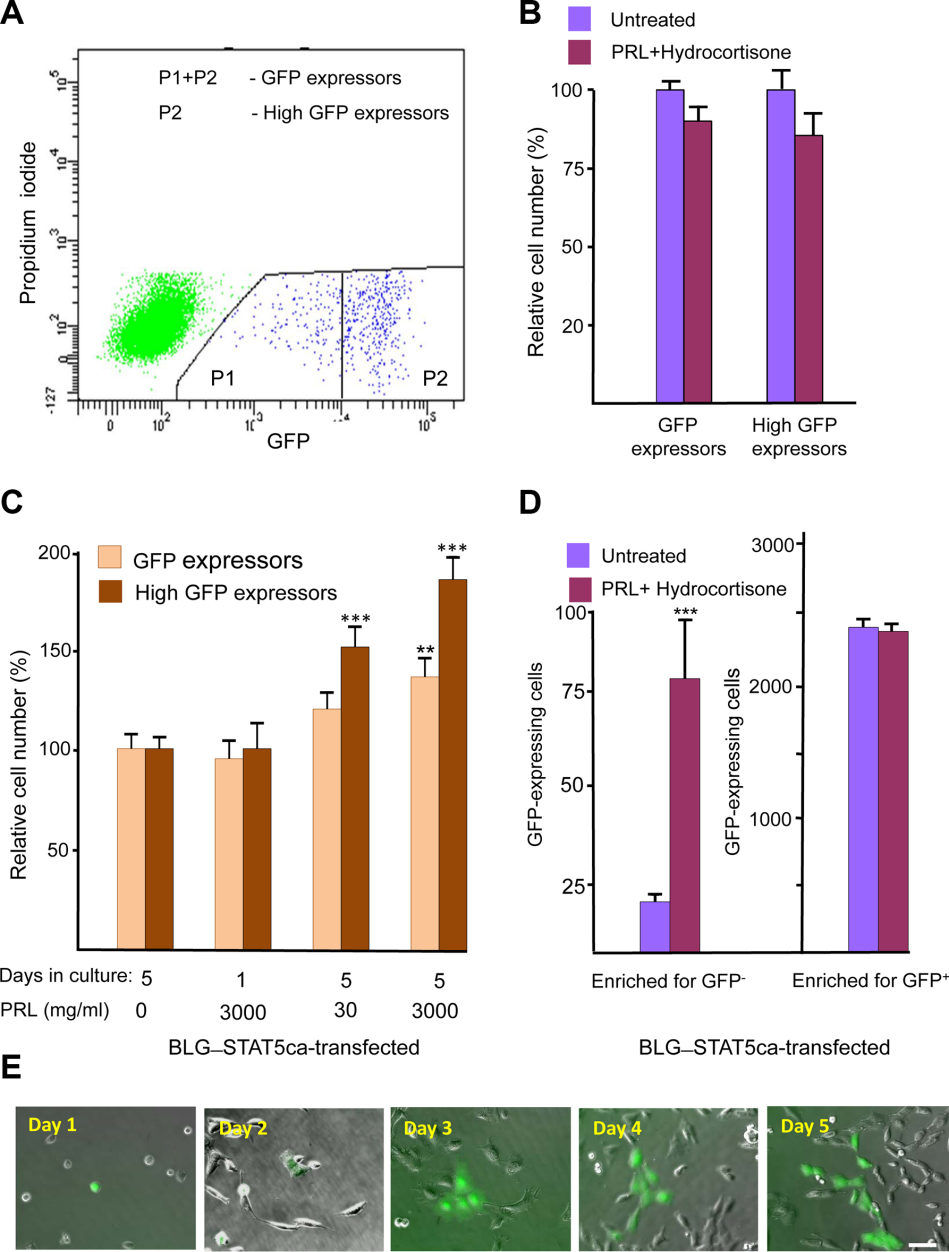
Identification of a scarce H2AX–GFP-expressing cell population which responds to lactogenic hormones only after transfection with forced-activated STAT5 (**A**) Representative FACS analysis of H2AX–GFP-expressing CID-9 cells. Non-transfected and BLG–STAT5ca-transfected cells share a similar pattern. The H2AX–GFP-expressing cells represent ~0.2% of the total cell population. (**B**) The number of H2AX–GFP-expressing cells is not affected by PRL and hydrocortisone supplementation to the insulin-containing growth medium in CID-9 cultures. (**C**) In cultures carrying the BLG–STAT5ca gene, PRL supplementation to insulin- and hydrocortisone-containing medium exerts a dose- and time-dependent effect on H2AX–GFP-expressing cells. (**D**) Supplementation of PRL and hydrocortisone to BLG–STAT5ca-transfected, H2AX–GFP-enriched cultures increases the number of GFP^+^ cells only in cultures initiated by GFP^−^ cells. Bars represent mean ± SEM of data from 3–5 independent experiments. (**E**) Development of a clone from H2AX–GFP-expressing cell. Bar = 50 μm.

An H2AX–GFP hybrid gene was constructed to follow H2AX promoter activity. A DNA fragment comprised of 960 bp upstream of the murine H2AX initiation site was linked to the GFP-coding sequence, introduced into the PCDNA3 expression vector and stably transfected into cultured mammary epithelial CID-9 cells (which express PRL and glucocorticoid receptor) as well as into CID-9 cells that were already carrying a forced-activated variant of the ovine Stat5, targeted for expression in the mammary gland by β-lactoglobulin (BLG) regulatory sequences and referred to as BLG–STAT5ca [12, 25]. Flow cytometry analysis performed after puromycin-based selection detected a remarkably low number of GFP-expressing cells in the non-transfected and BLG–STAT5ca-transfected cell cultures (~0.2% of total cell number, 4 independent transfections per culture). A subpopulation of high expressors was identified within the GFP-expressing cells of both cultures (Figure 1A). Supplementation of PRL and hydrocortisone to insulin-treated CID-9 cell cultures that did not carry the STAT5-based construct did not affect GFP-expressing cells (Figure 1B). In contrast, a significant (*P <* 0.05) increase in the number of total and high-GFP expressors followed supplementation of these hormones to the BLG–transfected cultures (Figure 1C). The individual effect of the STAT5 inducer PRL on the number of insulin- and hydrocortisone-treated GFP-expressing cells was time- and dose-dependent, and was more pronounced for the high-GFP expressors than for the total GFP-expressing cell population. Unless otherwise indicated, further studies were performed with BLG–STAT5ca-transfected cultures.

Is there a limit to the lactogenic hormone effect on H2AX–GFP-expressing cells? GFP^+^ and GFP^−^ cells from BLG–STAT5ca-transfected cultures were sorted and cultured separately in the presence or absence of PRL and hydrocortisone (Figure 1D). Only enriched cultures stemming from GFP^−^ cells responded to the hormonal supplementation, with a fivefold induction in the number of GFP-expressing cells. This indicated a limit to the effect of the lactogenic hormones on H2AX–GFP expression in these cells.

Of note, new individual GFP-expressing cells still appeared up to 5 days after culturing in lactogenic hormone-supplemented medium. Clone development is illustrated in Figure 1E. There was no indication of induced programmed cell death, as determined by TUNEL analysis, in the limited population of H2AX–GFP-expressing cells analyzed (*n* = 148), which did not exceed the 2.4% TUNEL-positive cells detected in the GFP^−^ cell population (Supplementary Figure S1).

In the next step, an enriched culture of H2AX–GFP-expressing cells was prepared from the cells that had undergone fluorescence-activated cell sorting (FACS) (Figure 1A) of the non-transfected and BLG–STAT5ca-transfected CID-9 cells. In these enriched cultures, the proportion of H2AX–GFP-expressing cells stabilized at ~2.0% of the total cell population (10-fold enrichment of the original transfected cells).

**Figure 2:**
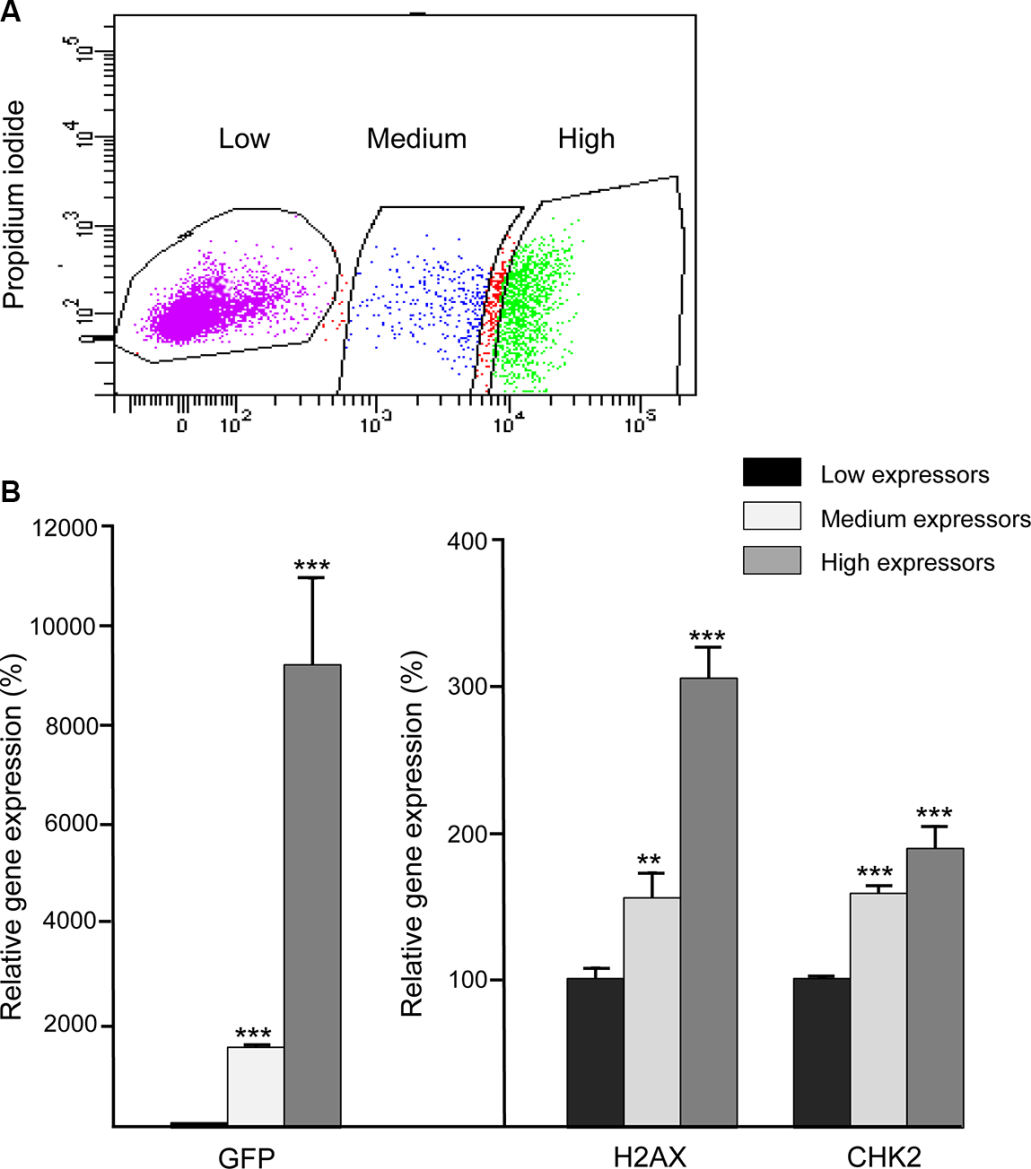
Expression patterns of the H2AX–GFP and native H2AX genes are correlated BLG–STAT5ca-transfected CID-9 cultures, enriched for 2% H2AX–GFP-expressing cells, were sorted for GFP expression and three populations, expressing increasing levels of the hybrid H2AX–GFP gene, were collected. RNA was extracted and gene expression was analyzed. GFP and H2AX gene expression was significantly (**P ≤ 0.01, ***P < 0.0001, *n* = 3) induced among cells that were defined as low, medium and high expressors by FACS. Bars represent mean ± SEM of data from 3 independent analyses.

To identify a putative correlation between H2AX promoter activity and endogenous H2AX expression levels, the H2AX–GFP-enriched BLG–STAT5ca-transfected cultures were sorted into three subpopulations according to their levels of GFP expression (Figure 2A). GFP RNA expression levels were 18- and 900-fold higher in the medium- and high-GFP expressors, respectively, compared to the low GFP-expressing cells, and correlated with the expression of the endogenous H2AX gene in these fractions (Figure 2B). Comparable significant (*P* < 0.05) changes in CHK2 expression, which is coactivated with H2AX by inducers of the DDR [35], were detected (Figure 2B), but no differences in BRCA1 expression could be observed among the three subpopulations (not shown).

The CID-9 cell culture is heterogeneous and contains luminal-like and basal-like cells [36]. They express the PRL receptor and glucocorticoid receptor (unpublished data). To morphologically distinguish the H2AX–GFP-expressing cells, their cytoplasmic and nuclear areas were compared to those of GFP^−^ cells in the enriched BLG–STAT5ca-transfected cultures. The GFP-expressing cells were identified by green fluorescence and DAPI-stained nuclei. The non-GFP-expressing cells were stained with hematoxylin and eosin (H&E) (data not shown). Cells of the GFP^+^ population were more heterogeneous in size than their GFP^−^ counterparts. Nevertheless, cell area peaked at 230 μm^2^ in the former, compared to 100 μm^2^ in the GFP^−^ population. On average, the area and nucleus size of the GFP-expressing cells were 1.8- and 1.4-fold larger, respectively, than those in their GFP^−^ counterparts.

Collectively, these results indicate that most of the CID-9 cells that are stably transfected with the H2AX–GFP construct maintain marginal H2AX promoter activity. Lactogenic hormone supplementation induced H2AX promoter activity and endogenous H2AX gene expression in a limited number of distinct cells, depending on the ability of the culture to generate a hyperactivated STAT5 signal.

### Characterization of H2AX–GFP-expressing cells in the mammary gland of transgenic mice

**Figure 3:**
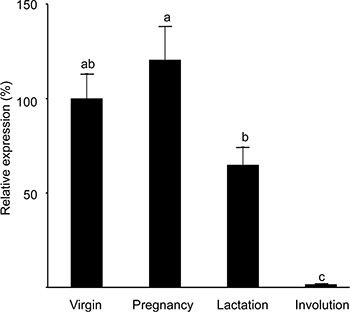
Expression of the H2AX–GFP transgene peaks at pregnancy and is significantly reduced in the involuting gland RNA was extracted from the mammary glands (#4) of transgenic mice of strain #102 at each period of the reproductive cycle and expression levels of the H2AX–GFP transgene and H2AX gene were determined by RT-PCR analysis. Bars represent mean ± SEM of data obtained from 9–11 mice in each physiological state. Different letters represent significant (*P ≤* 0.05) difference. The results were confirmed in the mammary gland of an additional line (#88).

The putative presence of a distinct cell population with high H2AX promoter activity in the mammary gland was studied in transgenic mice expressing the H2AX–GFP hybrid gene. Two lines (#88 and #102) expressing the transgene at detectable levels in their mammary glands were employed. In both lines, the number of GFP-expressing cells in the mammary gland of virgin females was lower by an order of magnitude than that in the stably transfected CID-9 cell population (100–200 cell/10^6^ mammary epithelial cell). Detailed experiments, including double-transgenic ones, were performed with line #102 and confirmed with mice of line #88. Expression of H2AX–GFP was higher in the pregnant gland (Figure 3). It declined sharply during lactation and involution, thus resembling the expression of the endogenous H2AX gene *in vitro* under conditions that mimic the parity cycle [25].

**Figure 4:**
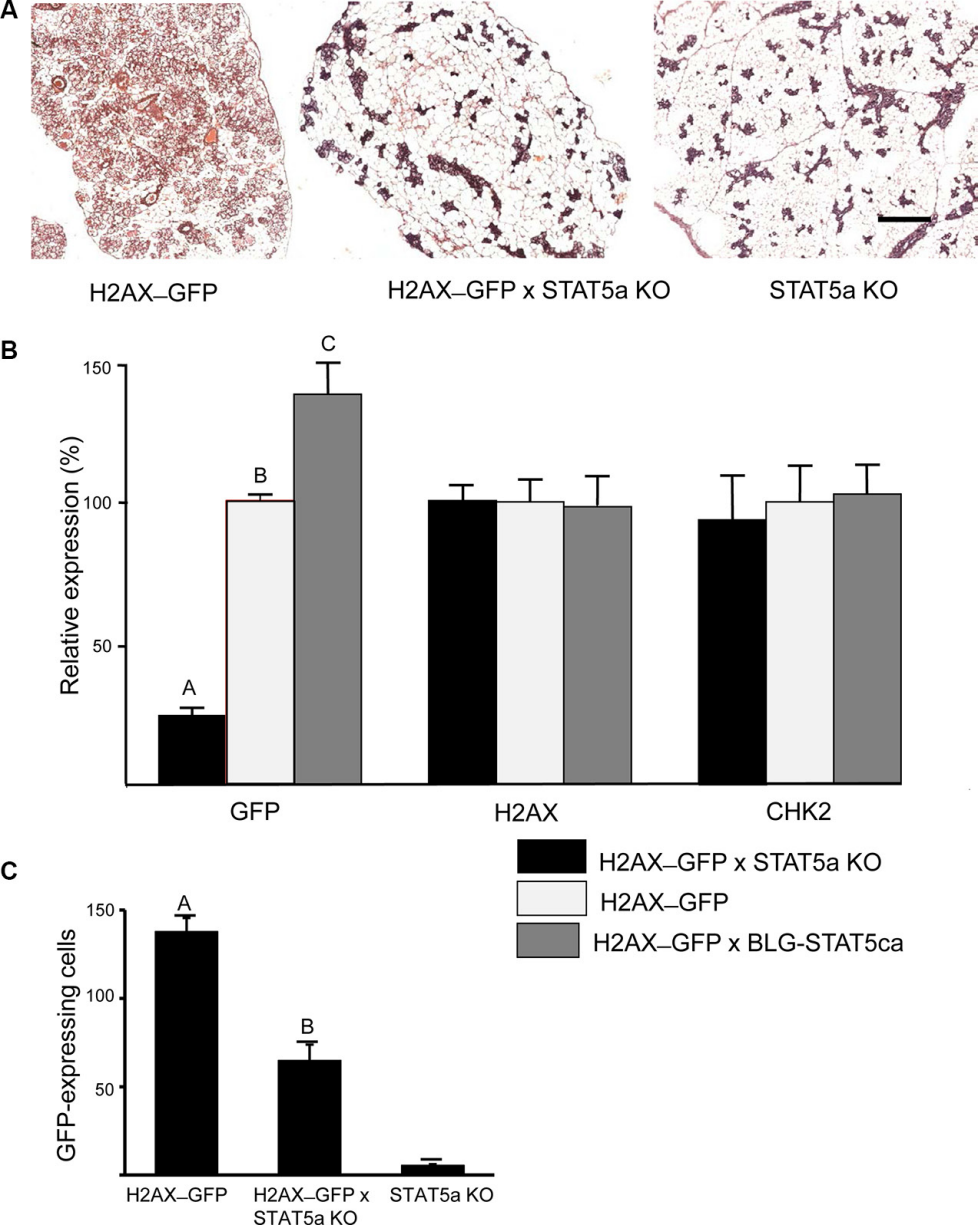
H2AX–GFP expression is abrogated in Stat5a-null mice and enhanced in the presence of forced-activated STAT5 Genetically modified mice expressing the H2AX–GFP gene on the genetic background of STAT5a^−/−^ (KO) or BLG–STAT5ca (forced-activated STAT5) were generated. (**A**) STAT5a KO/H2AX–GFP mice maintained a poorly developed gland on days 17–18 of pregnancy. (**B**) GFP expression was significantly (*P ≤* 0.05) reduced or increased in the mammary gland of H2AX–GFP mice expressing the KO or forced-activated STAT5, respectively. Lack of effect on the native gene confirms the scarcity of the H2AX–GFP-expressing cells (*n* = 7–9 mice per group). (**C**) Expression pattern of the H2AX–GFP gene correlates with the number of H2AX–GFP-expressing cells (see Figure 5B for comparison). Mice expressing the STAT5-KO gene, but not H2AX, served as negative controls for GFP detection. Different letters indicate a significant (*P ≤* 0.05) difference; *n* = 4 mice in each group.

The involvement of STAT5 in mediating H2AX promoter activity was studied in pregnant females on the altered genetic background of the Stat5a-null allele, or in the presence of the BLG–STAT5ca transgene [12] (Figure 4). The reportedly underdeveloped mammary gland phenotype that characterizes the Stat5a-null mice in their first pregnancy [11] was recovered in the H2AX–GFP/Stat5a knockout (KO) mice (Figure 4A). In these mice, lack of Stat5a expression resulted in a significant (*P <* 0.01) eightfold decrease in expression of the H2AX–GFP hybrid gene (Figure 4B), which mirrored the decrease in the number of GFP^+^ cells (Figure 4C). In contrast, forced activation of STAT5 resulted in increased (by about 42%) H2AX–GFP expression. These changes were not associated with detectable differences in expression of the endogenous H2AX or CHK2 genes in the crude extract of the mammary gland (Figure 4B) due to the masking effect of transcripts from most cells that do not express GFP, suggesting a specific effect of the altered STAT5 activity/expression on the rare and distinct H2AX–GFP-expressing cells. Indeed, it was only in the enriched GFP^+^ cultures (Figure 2), which contained 2–3 orders of magnitude more H2AX–GFP-expressing cells, that a correlation between promoter activity and native gene expression could be demonstrated.

**Figure 5:**
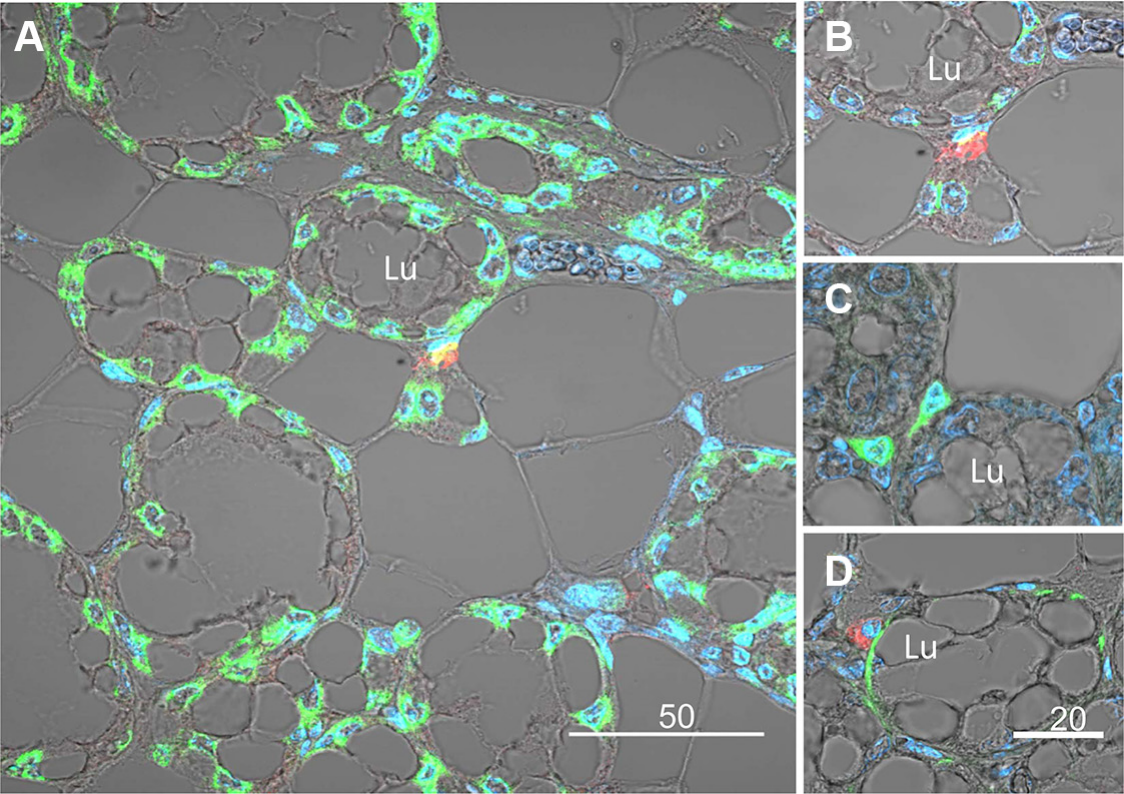
Immunofluorescence analysis and confocal microscopy demonstrating expression of the H2AX–GFP gene in basal epithelial cells composing the external layer of the lobuloalveolar structures of the pregnant mammary gland (**A**) Compared to the native H2AX that is expressed in most epithelial cells (green), the H2AX–GFP transgene (red) is exclusively expressed in rare basal cells. Nuclei are stained with DAPI. (**B**) Larger magnification of the H2AX–GFP-expressing cells. (**C**) Analysis of the H2AX–GFP-expressing cells (green), confirming basal location. (**D**) Colocalization of α-smooth muscle actin (αSMA, green) and H2AX–GFP (red) expression in basal cells of the mammary lobuloalveolar structures. Note that even though the colors can be distinct, the cells share the same nuclei. Lu, lumen.

**Figure 6:**
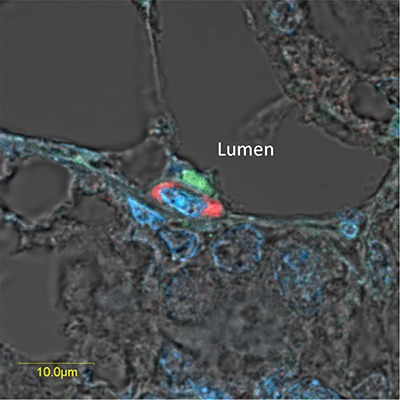
Overexpressed pSTAT5 and H2AX-driven GFP are located in neighboring cells of the luminal and basal compartments, respectively, in the lobuloalveolar structure of the pregnant mammary gland Immunofluorescence analysis and confocal microscopy localizing neighboring epithelial cells that express highly active (phosphorylated) STAT5a (green) and H2AX-driven GFP (red) in the luminal and basal compartments, respectively, of a lubuloalveolar structure in the pregnant mammary gland. Exposure of the red-stained cells was fine-tuned to show only the highly expressing cells.

The morphology of the lobuloalveolar structures in the pregnant mouse mammary gland is demonstrated in H&E-stained paraffin sections (Supplementary Figure S2). Further experiments were performed to morphologically localize the H2AX–GFP-expressing cells in the mammary tissue by confocal microscopy of immunofluorescence-stained sections of pregnant female mice (Figure 5). These analyses, exemplified by representative images in Figure 5A, 5B, confirmed the scarcity of the H2AX–GFP-expressing cells, which were individually located in the basal/superbasal layer of the lobuloalveoli (Figure 5A–5C). The unexpected basal location of the H2AX–GFP cell population diverged from the well-established involvement of STAT5 in luminal epithelial cell differentiation [10]. It was therefore confirmed by colocalization of H2AX–GFP and α-smooth muscle actin (α-SMA) expression, the latter being a marker for contractile basal myoepithelial cells (Figure 5D). In contrast, H2AX–GFP-stained cells could be distinguished from the CK18-stained luminal cells (Supplementary Figure S3). Importantly, the basally located H2AX–GFP-expressing cells did not express phosphorylated STAT5, which was detected exclusively in their neighboring luminal cells (Figure 6).

**Figure 7:**
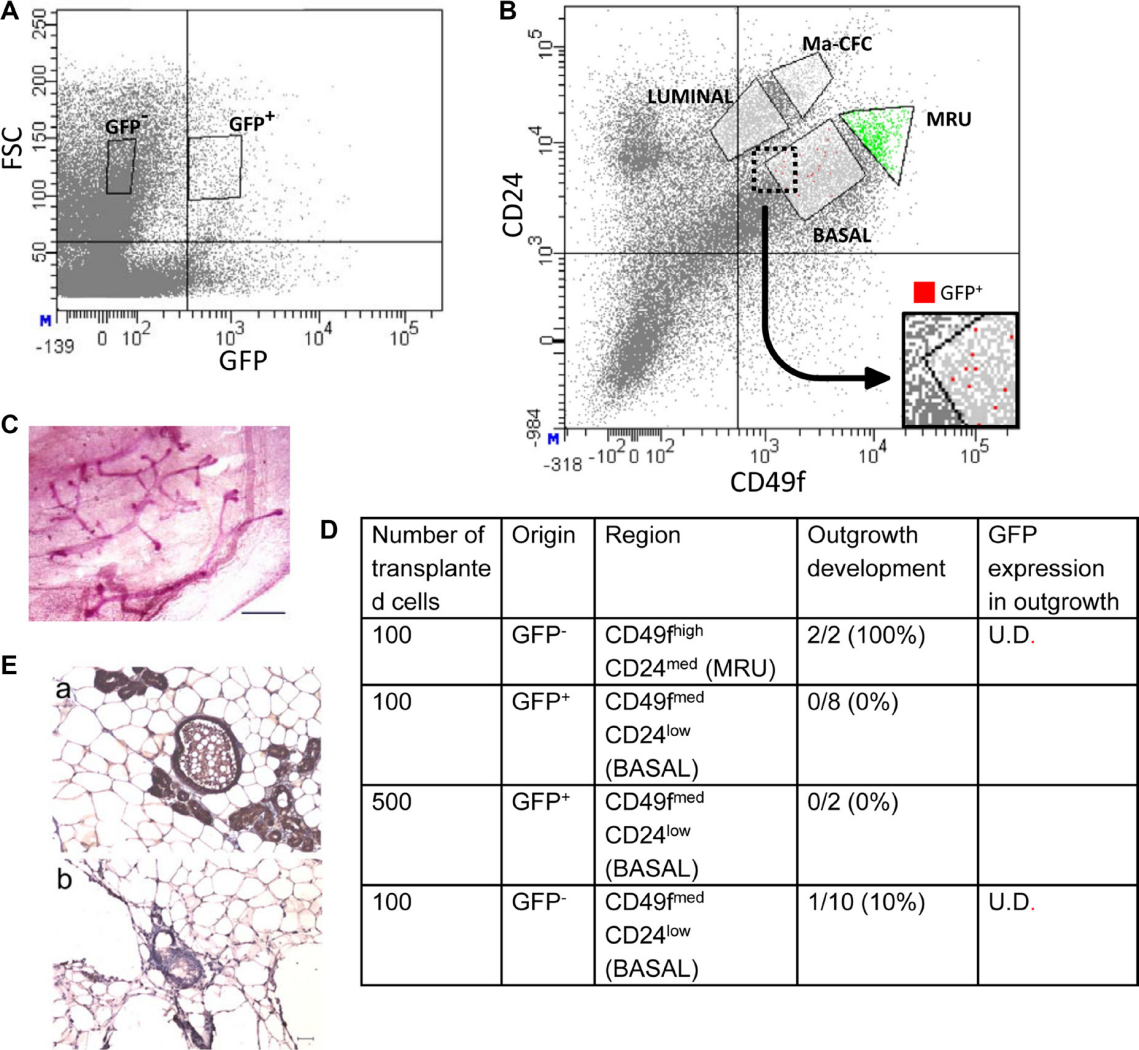
FACS analysis and cell transplantation indicate that H2AX–GFP-expressing cells acquire basal, but not stem-cell properties (**A**) Selection of GFP^+^ and GFP^−^ cells by FACS. FSC, forward scatter. (**B**) Coanalysis of CD24 and CD49f in the sorting procedure allocates H2AX–GFP cells to the basal compartment. Ma-CFC, mammary colony-forming cells; MRU, mammary repopulating unit. (**C**, **D**) Upon cell transplantation into the cleared mammary fat pad of host mice, H2AX–GFP cells do not generate outgrowths, in contrast to MRUs. (**E**) MRU cells from H2AX–GFP-transgenic mice do not express GFP (b) as compared to control mice (a) with ubiquitous GFP expression (kindly provided by Prof. Ilan Tsarfati, Tel Aviv University, Israel). Immunocytochemical analysis with DAB as a marker. Bar = 50 μm. U.D., undetectable.

Further confirmation of the basal origin of the rare H2AX–GFP-expressing epithelial cells was obtained by FACS analysis of Lin^−^ mammary epithelial cells (Figure 7), using the GFP^−^ cell as a negative control (Figure 7A). CD49f and CD24 have been previously used to enrich mouse epithelial cell populations making up the mammary gland cell hierarchy [8, 37, 38]. According to their expression, four subpopulations were identified and sorted from both GFP^+^ and the GFP^−^ cell populations (Figure 7B): (i) a population enriched in MRUs [37–40]; (ii) a basal population encompassing the differentiated myoepithelial cells and upstream progenitors (BASAL) [8]; (iii) unipotent luminal-restricted progenitors, termed mammary colony forming cells, and (iv) fully committed mammary luminal cells (LUMINAL) [37, 38].

The rare H2AX–GFP cell population was localized to the CD49f^med^CD24^low^ region of the plot (Figure 7B), indicating a basal origin. Control transplantation of 100 GFP^−^ cells of the MRU fraction (CD49f^high^CD24^med^) into the recipient's cleared fat pad resulted in the development of outgrowths in all glands (Figure 7C, 7D), thus confirming previous reports regarding their location and the number of cells required to repopulate the cleared fat pad [38]. In contrast, no outgrowth developed from the basally originated CD49f^med^CD24^low^ GFP^+^cells, even when 500 cells were transplanted. Out of the 10 glands transplanted with GFP^−^ basal cells, only a single outgrowth developed. Importantly, no detectable GFP expression could be determined in any of the developing outgrowths (Figure 7E), indicating that transplantation into a foreign environment is not sufficient to initiate GFP expression in non-expressing cells.

### H2AX promoter methylation is involved in regulating H2AX expression

**Figure 8:**
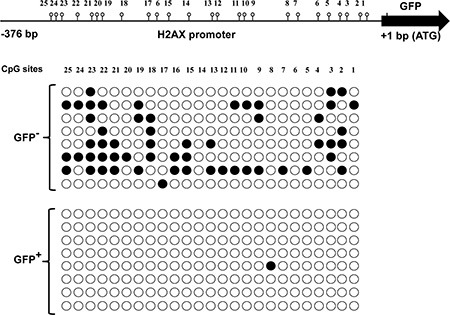
Methylated vs. non-methylated H2AX promoter distinguishes non-H2AX–GFP-expressing cells from their H2AX–GFP-expressing counterparts The methylation status of CpG sites was determined by bisulfite sequencing (●, methylated; ○, non-methylated).

The mechanism behind the limited H2AX promoter activity in most basal mammary cells and the partial release of H2AX–GFP expression in a few of these cells neighboring the luminal cells with hyper STAT5 activity were further studied, focusing on H2AX promoter methylation. Here, CID-9 cultures that were enriched by FACS for constitutively H2AX–GFP-expressing- or non-expressing cells were used. The methylation status of 25 individual GpG sites in the proximal H2AX promoter region (376 bp upstream of the transcription initiation site of the GFP gene) was analyzed by the bisulfite genomic sequencing method. DNA sequencing of eight individual clones from the enriched cultures of H2AX–GFP^+^ and H2AX–GFP^−^ cells revealed almost no CpG methylation at the H2AX promoter in H2AX–GFP^+^ cells, but a certain degree of methylation in the H2AX–GFP^−^ cells (Figure 8). Most CpG sites (excluding sites 6, 8 and 14) of the H2AX–GFP^−^ cells were methylated in 12.5 to 75% of the clones: site 23 was methylated in 75% of the clones, site 22 in 62% of the clones, and sites 2, 3 and 18 that were methylated in 50% of the clones. In contrast, only a single methylation was detected out of 200 CpG candidates in the GFP^+^ cells.

**Figure 9:**
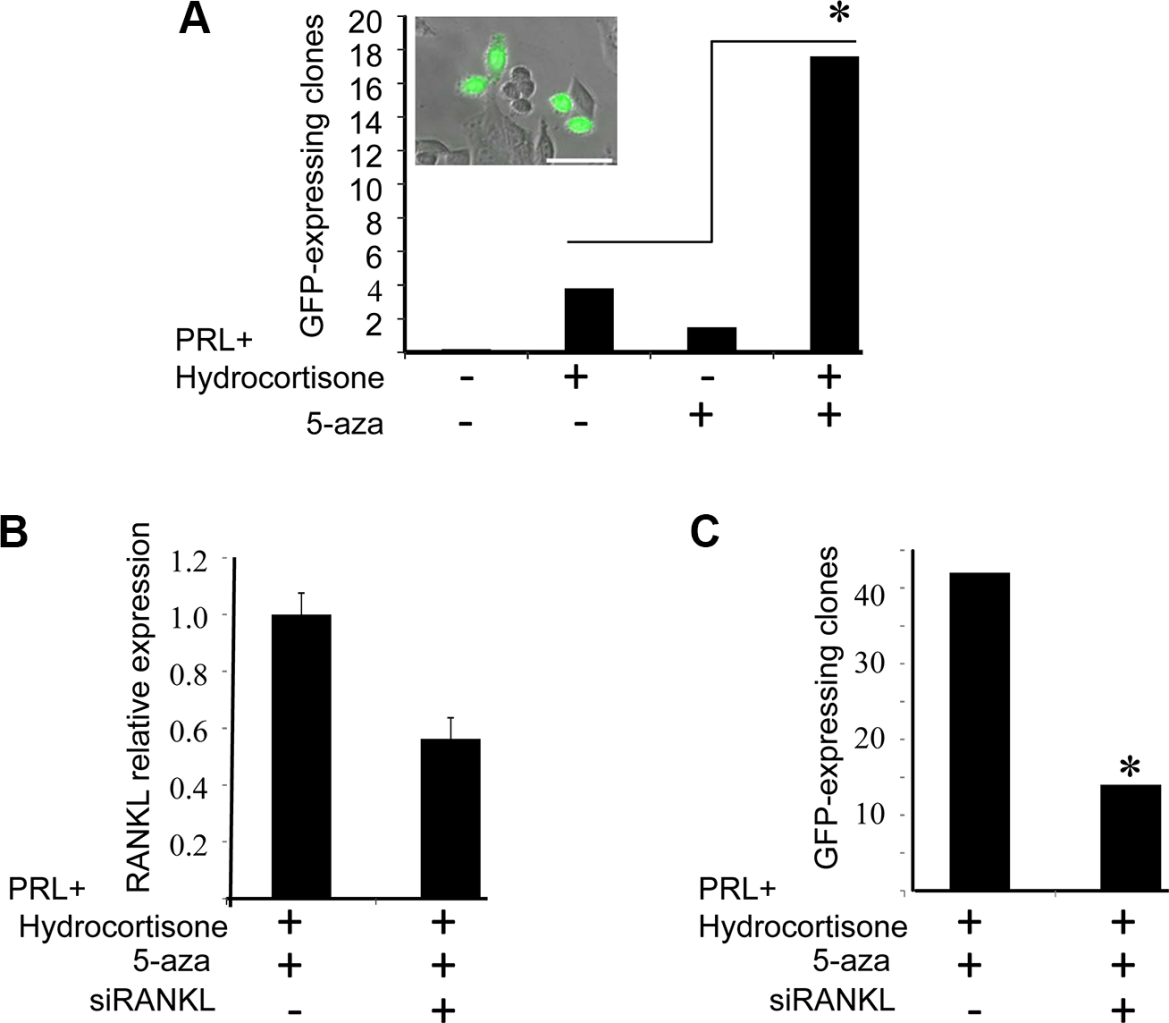
Supplementation of 5-azacytidine (5-aza) increases the number of H2AX–GFP-expressing clones via RANKL paracrine signaling (**A**) The effect of 5-aza on the number of visible H2AX–GFP clones (≥ 1 cell) was monitored in BLG-STAT5/H2AX–GFP-transfected cultures on day 4 of treatment, in the presence or absence of PRL and hydrocortisone. Bars represent the number of visible H2AX–GFP cells/clones (GFP^+^) per 100,000 seeded cells. The clones were monitored in 108–204 wells (96-well plate) seeded with 1000 cells each. Chi square values were calculated and significant differences (*P* < 0.001), indicated by an asterisk, were determined using Pearson test. Inset: two GFP-expressing clones. Bar = 50 μm. (**B**) The effect of RANKL siRNA transfection on RANKL expression was determined by real-time PCR after 3 days of treatment with PRL, hydrocortisone and 5-aza. Bars represent an average of two independent transfections with range determination of RANKL, or non-specific siRNA. (**C**) RANKL siRNA transfection suppressed the number of GFP^+^ cells/clones under the conditions elaborated in (B). Bars represent the number of GFP^+^ clones per 50,000 seeded cells, monitored in 28 or 48 wells, seeded with 2000 cells each and monitored on day 4 of culture. Chi square values were calculated and significant differences (*P* < 0.01), indicated by an asterisk, were determined using Pearson test.

To further confirm promoter methylation's involvement in regulating H2AX–GFP expression, BLG–STAT5ca-transfected CID-9 cultures (Figure 1) were exposed to the methylation inhibitor 5-azacytidine (5-aza). In the presence or absence of lactogenic hormones, cultured cells were either treated or not treated with 5-aza for 3 days [41] and H2AX–GFP-expressing clones were monitored in three separate experiments (Figure 9A). The supplemented dosage (2 μg/ml) and experimental time course were previously calibrated for an optimal effect on H2AX–GFP expression with minimal toxic effect on the cells. H2AX–GFP-expressing clones were not observed in the untreated cells. In contrast, a detectable number of GFP^+^ clones was observed after lactogenic hormone or 5-aza administration. Importantly, the major effect of 5-aza was observed on the hormonally treated cells. In these cells, a significant (*P* ≤ 0.001) increase in the number of H2AX–GFP-expressing clones followed 5-aza administration, enhancing the number of lactogenic hormone-treated clones 4.5-fold. Since no H2AX–GFP-expressing clones were observed in the untreated cell cultures, they could not be included in the formal statistical analysis.

### Evidence for RANKL involvement in transducing pSTAT5 signal

An important question that remained to be addressed related to the identity of the factor(s) that transduce the signal from luminal cells with high STAT5 activity to the basal cells. Based on STAT5 involvement in RANKL-mediated progesterone paracrine signaling [42], we analyzed the effect of RANKL siRNA on the number of visible H2AX–GFP clones generated by BLG–STAT5ca/H2AX–GFP-transfected CID-9 cells. These cells were transfected with RANKL siRNA and treated with lactogenic hormones and 5-aza for 3 days to allow clone generation and visible GFP expression. Transfection of RANKL siRNA reduced RANKL expression by 45% after 3 days of culture (Figure 9B), and the number of visible H2AX–GFP-expressing clones was reduced by 66%. This observation supports the involvement of RANKL in mediating the generation of H2AX–GFP expression.

## DISCUSSION

Forced activation of STAT5 results in parity-dependent development of tumors in the mammary glands of the transgenic mice [22, 23]. Under pregnancy-like, in-vitro conditions, CID-9 mammary epithelial cultures transfected with the forced-activated STAT5 have higher expression of the H2AX gene compared to their non-transfected counterparts [25]. To elucidate the involved mechanism and the distinct cell populations that might be subjected to altered H2AX gene expression, a GFP-linked H2AX promoter was introduced into the genome of mammary CID-9 epithelial cells and transgenic mice.

An extremely low proportion of H2AX–GFP-expressing cells characterized non-transfected and BLG–STAT5ca-transfected CID-9 cell cultures, as well as transgenic mouse mammary glands. Based on the presented data, this key observation may reflect suppression of H2AX–GFP expression by CpG methylation, which suppresses H2AX–GFP expression in most cells. The correlation between the expression levels of H2AX–GFP and endogenous H2AX suggests that detectable GFP expression marks mammary epithelial cells with very high expression of the endogenous gene. Such high H2AX expression could make this rare population prone to tumorigenesis [33, 34, 43], warranting its further characterization—especially of its STAT5-dependent regulation.

What are the characteristics of the H2AX–GFP-expressing cells that distinguish them from the general mammary cell population? A morphological difference was observed in culture. Within the CID-9 heterogeneous population [36], the area of the nucleus and cytoplasm of H2AX–GFP-expressing cells was larger than in their non-expressing counterparts. In addition, the rapid and high response to 5-aza suggests a proliferation-independent inductive effect of STAT5-mediated lactogenic hormone on the number of H2AX–GFP-expressing cells. Better distinction of this population in the mammary gland was obtained by immunofluorescence detection and flow cytometry analysis (FACS), followed by cell transplantation of dispersed mammary cells from transgenic mice expressing the H2AX–GFP promoter. These complementary analyses indicated that the H2AX–GFP-expressing cells are located in the outer epithelial layer that surrounds the lumen, express the basal marker α-SMA (but not the luminal markers CK18 or Stat5a), and fall within the CD24^low^CD49f^med-low^ parameters of basal mammary epithelial cells in flow cytometry [44]. The tempting speculation that they represent the basally located stem-cell population that renews the mammary gland in subsequent pregnancies was dismissed due to their lower CD24 expression compared to the MRUs [44], and especially their lack of self-renewing potential upon transplantation into the cleared mammary fat pad of female hosts. Indeed, the H2AX–GFP-expressing cells also do not acquire self-renewing potential, as suggested for part of the basal cell population [8].

Thus, the H2AX–GFP-expressing cells seem to constitute a rare basal population that peaks at pregnancy and depends strictly on luminal STAT5 expression. Immunofluorescence analysis demonstrated close morphological proximity between these cells and the luminal cells with high STAT5 activity. Taken together, it is suggested that H2AX promoter activation and probably endogenous gene expression in the basal mammary population are affected by signals from adjacent luminal cells with high STAT5 activity. At present, little is known about the specific regulation of basal cell proliferation. Here, PR-mediated paracrine signaling may be shared [45, 46]. Progesterone initiates the mammary secretory process via PR, leading to RANKL-mediated Elf5 expression in PR^−^ luminal progenitor cells [47] with Stat5a as a cofactor [42]. The RANKL-mediated pathway is also active in the progesterone effect on the basally located stem cells during pregnancy and luteal cycles [48, 49].

Interference of RANKL expression by siRNA demonstrated the involvement of RANKL-mediated paracrine signaling in transducing the STAT5-dependent effect from the luminal cells to their neighboring basal ones, ultimately leading to an increase in the number of H2AX–GFP-expressing cells. The proposed model of an indirect STAT5 effect mediated by RANKL, and possibly other transducers, on basal cells reconciles previous reports ruling out a direct lactogenic hormone-induced Stat5a effect on this cell population [10]. A most recent report demonstrating that in late pregnancy, RANKL inhibits PRL-induced lactogenesis and terminal differentiation by interfering with Stat5 activation, but also expands basal cell population, supports our finding [50].

Most interestingly, a signaling pathway that works in the opposite direction has been recently documented [9]. It involves secretion of NRG1 (neuregulin, heregulin-α) from p63-expressing basal cells, which induces ERBB4-activating pSTAT5 in the neighboring luminal cells. Those data suggested that the NRG1/ERBB4/STAT5 signal is essential for luminal mammary gland progenitor maturation and therefore for mammary gland development.

Which intercellular mechanism that is relevant to H2AX–GFP expression responds to STAT5-mediated signaling in the basal mammary cells? The suppression of H2AX promoter activity in most cells, as well as its partial release, were further addressed, focusing on promoter methylation. In mammals, DNA methylation is a key epigenetic modification that is essential for normal genome regulation and development and is generally inversely correlated with gene expression (reviewed by [48]). Methylation, and especially promoter methylation [51], stabilizes the silent state of genes by either blocking DNA-binding transcription factors, or recruiting methyl-CpG-binding domain proteins, which favor the formation of heterochromatin [48]. The impact of methylation has been demonstrated for a variety of promoters and is restricted to genes containing CpG dinucleotides: even low numbers of 5′-CG-3′ dinucleotides, e.g., four in the human TNFα promoter, may suffice to elicit complete inactivation. Conversely, the LTR-MMTV promoter, which is devoid of 5′-CG-3′ dinucleotides, does not respond to 5′-CG-3′ methylation and its activity continues unabated [52].

The relevance of methylation to limiting the number of H2AX–GFP-expressing cells in the transfected cultures and in the mammary gland of transgenic mice was confirmed by bisulfite genomic sequencing method. The analysis showed the absence of methylated CpG sites in the proximal H2AX promoter in DNA extracted from stably expressing, highly enriched H2AX–GFP cells. In contrast, 22 out of 25 candidate sites within the same DNA region extracted from their non-expressing counterparts were methylated in 12.5–75% of the clones. Interestingly, the highly methylated region (−283 to −293 bp) encompassed CpG sites 19–21, which contain high-confidence, in silico-defined, cis-regulatory binding sites for the E2F that regulates transcription of many cell-cycle genes. In the retinoblastoma (Rb-1) tumor-suppressor gene promoter, methylated E2F sites become refractory to binding by E2F factors [53]. Relaxed inhibition of E2F activity via demethylation of its binding site can potentially enhance H2AX promoter activity and activate uncontrolled H2AX–GFP^+^ cell proliferation [33]. Moreover, the ER/PR paracrine signaling also involves methylation that defines endocrine sensitivity to cancer. It has been shown that the 5′ region of the ESR1 gene is methylated in ER^−^ breast cancer cell lines [54]. Conversely, cells with increased ER levels, such as LTED cells, contain hypomethylated CpG islands in the ESR1 promoter [55, 56]. In addition, ER enhancer hypomethylation is critical for the transition from normal mammary epithelial cells to endocrine-responsive ER cancer cells [57]. Relating hypomethylation to cancer in the context of the H2AX promoter may complement previous observations linking H2AX overexpression and fibroblast immortality [33, 34], and position H2AX overexpression as a putative contributor to STAT5-induced carcinogenesis.

**Figure 10:**
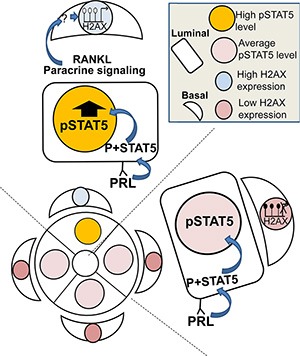
Illustration of the working hypothesis suggesting that deregulated STAT5 activity is transduced from luminal to basal mammary cells and causes H2AX promoter activation Paracrine signaling via RANKL (and possibly other transducers) from rare luminal cells with highly activated STAT5 induces demethylation and activation of the H2AX promoter in their neighboring basal cells. This includes the involvement of yet undetermined transcription factors. Note: a direct effect of STAT5 overexpression in a single luminal cell on RANKL secretion, and the mechanism by which RANKL affects demethylation of the H2AX promoter in neighboring basal cells have yet to be demonstrated.

A question that still remained to be answered was related to the specificity of the H2AX–GFP-expressing cells for high expression of the individual H2AX gene. *In-vitro* analysis suggested that these cells may also incorporate high expression of other selected genes, such as CHK1. Nevertheless, the fact that 5-aza administration increased the number of H2AX–GFP-expressing cells in a lactogenic hormone-dependent, STAT5-mediated manner supports the hypothesis of methylation as a silencing mechanism of H2AX (and possibly CHK1) expression, and of STAT5 hyperactivity as an indirect inducer of demethylation (Figure 10).

To date, the role of STAT5 in the mammary gland has been thought to be limited to affecting development and function of the luminal lineage. The data presented here suggest a novel mechanism for a RANKL-mediated paracrine STAT5 effect that incorporates increased luminal STAT5 signal to execute H2AX promoter activity, via its demethylation in neighboring basal cells. Imbalanced H2AX expression during late pregnancy, the period with the highest Stat5a activity, may contribute to STAT5′s latent tumorigenic effect in the mammary gland.

## MATERIALS AND METHODS

### Construction of recombinant H2AX–GFP gene

A 1425-bp fragment containing the H2AX gene promoter was amplified from mouse genomic DNA. This fragment was used as a template for a nested PCR (1040-bp product), aimed at introducing the NdeI and NheI restriction sites at the distal and proximal ends of the H2AX promoter, respectively (all primers are given in Supplementary Table 1). Restriction of this product with the above enzymes resulted in a 1003-bp fragment which was inserted into the AseI–NheI sites of the pEGFP-C3 vector (Clontech, Mountain View, CA). From this vector, a 1968-bp H2AX–GFP, KpnI–XhoI, fragment was excised and subcloned into the respective sites of the pCDNA3 vector (Life Technologies, Carlsbad, CA). The resulting chimeric H2AX–GFP fragment consisted of 960 bp from the H2AX promoter (including its TATA-box at the original positions: −61 to −64 relative to ATG), followed by a 6-bp common NheI restriction sequence, 11-bp sequences upstream of the EGFP start codon and the full EGFP coding region terminated by 263 bp of the 3′-untranslated region, including two poly(A) sites.

### Generation of H2AX–GFP-transgenic mice

Mice used in this study were of the FBV/N strain. A 2184-bp ZraI–XhoI fragment containing H2AX–GFP sequences was excised from the pCDNA3 vector that contained the chimeric gene, purified on an agarose gel and microinjected into the mouse's pronuclei as described previously [58]. Transgenic mice were identified by PCR analysis of tail biopsies digested with Direct PCR Lysis Reagent (Viagen Biotech, Los Angeles, CA) using primers located at the 3′ end of the H2AX promoter and the 5′ end of GFP (Supplementary Table 1). To monitor pregnancy, presence of a vaginal plug marked day 1. All animals used in this study were treated humanely. Study protocols were in compliance with the regulations of the Israeli Ministry of Health and local institutional policies.

### CID-9 cell culture and transfections, 5-aza administration

CID-9 cells were grown in Dulbecco's modified Eagle medium (DMEM):F12 (1:1 Gibco-BRL, Paisely, UK) containing 5% fetal calf serum (FCS), glutamine (365 μg/ml, Biological Industries, Beit Haemek, Israel), gentamicin (50 μg/ml, Biological Industries) and insulin (5 μg/ml, Sigma, St. Louis, MO). Unless otherwise indicated, the effect of PRL and hydrocortisone supplementation was studied in a six-well culture plate (Corning, NY) for 5 days in DMEM:F12 containing 5% FCS, insulin (5 mg/ml), hydrocortisone (3 mg/ml, Sigma), and ovine PRL (3 mg/ml, provided by the NIDDK program). Stable transfections of the H2AX–GFP gene construct into non-transfected CID-9 cells, or BLG–CID9ca-transfected cells carrying a constitutively active STAT5 gene directed for expression in the mammary gland by BLG regulatory sequences (BLG–STAT5ca) [12, 25], were carried out with lipofectamine reagent (Gibco-BRL, Gaithersburg, MD) and pBABE plasmid [59]. Selection was performed with puromycin (2 μg/ml, Sigma). Surviving colonies were pooled and stocks were grown. To examine the effect of 5-aza, 1000 cell/well were cultured in 96-well plates, and freshly prepared 5-aza (2 μg/ml) was supplemented daily for 3 days to insulin- and FCS-treated cells that were either supplemented or not with lactogenic hormones. The number of H2AX–GFP-expressing clones was monitored on day 4 of the experiment.

Transient transfection of RANKL siRNA (mouse, Santa Cruz Biotechnology, Dallas, TX) was performed according to the manufacturer's protocol and cells were supplemented for 3 days with DMEM:F12 containing FCS, insulin, hydrocortisone, PRL and 5-aza. The number of H2AX–GFP-expressing clones was monitored on day 4 of the experiment as described for 5-aza analysis, except for the number of seeded cells, which was 2000/well.

### Preparation of single mammary cell suspension, mammary fat-pad clearing and cell transplantation

Single-cell suspension was prepared from #4 mammary glands. The tissue was excised and minced with fine scissors into 1- to 3 mm^3^ pieces. These pieces were digested at 37^°^C for 3 h in slowly shaken 50 ml conical tubes containing 10 ml DMEM:F12 medium supplemented with 5% FCS, type II collagenase (300 U/ml, Worthington, Lakewood, NJ), hyaluronidase (100 U/ml), insulin (5 mg/ml) and hydrocortisone (1 mg/ml), all from Sigma. The resulting organoids were washed in HBSS (Biological Industries) containing 5% FCS (HF solution) and treated for 3 min, first with trypsin-EDTA solution (Biological Industries) and then with dispase enzymatic solution (50 caseinolytic unit/ml, BD Biosciences, Bedford, MA) containing DNAse-I (0.125 mg/ml, Worthington). The dissociated cells were washed, resuspended in HF solution and separated from tissue debris and cell aggregates by filtration through a metal mesh, followed by a cell strainer (BD Falcon, Bedford, MA) with pores of 70 μm and 40 μm diameter, respectively.

The surgical techniques used to clear the mammary epithelium of the fat pads of 3-week-old host mice were as previously described ([60] and references therein). In brief, the mice were anesthetized, and the clearing procedure was performed immediately before insertion of the transplanted cell suspensions. Complete clearing of the mammary glands was confirmed by Carmine red staining of the removed tissue, showing clear, unindaveded margins. Cell suspensions were implanted in 10 μl volumes of Matrigel (Collaborative Bio-Medical Products, Bedford, MA) diluted 1:1 in DMEM:F12 with a Hamilton syringe equipped with a 21-gauge needle.

### Whole-mount and histological analyses, immunostaining and TUNEL assays

For whole-mount examination, the transplanted mammary fat pads were excised from sacrificed mice and fixed on glass slides with 4% paraformaldehyde containing 1% sucrose for 2 h at room temperature. Whole mounts were washed with PBS and stained with Carmine-alum (Sigma) for 3 days at room temperature as previously described [60]. Whole mounts were dehydrated with ethanol, cleared in K-clear reagent (Kaltek, Padova, Italy) overnight at room temperature, and visualized and photographed using a binocular (Olympus SZX16, Tokyo, Japan) equipped with CellSens standard 1.4 software (Olympus). The tissues were then embedded in paraffin blocks for further analyses.

GFP-expressing cells were directly visualized in culture. Immunostaining of cultured cells was performed using eight-chamber cell culture slides (SPL Life Science Co., Pocheon, Korea). Cells were fixed with 4% paraformaldehyde, washed with PBS and treated with 0.5% Triton X-100 (BDH, Poole, England) for 5 min. Following overnight incubation in blocking solution (2% goat serum and 1% bovine serum albumin in PBS) at 4°C, the fixed cultures were reacted with primary antibodies for 1 h at room temperature and then overnight at 4°C. Incubation with secondary antibodies proceeded for 1 h at room temperature, and nuclei were stained with DAPI (Qbiogen, Irvine, CA). The antibodies and their dilutions are listed in Supplementary Table 2.

For mammary tissue immunostaining, biopsies were fixed in Bouin's solution, dehydrated in a graded ethanol series (50% to 100%), cleared in xylene and embedded in paraffin. Immunostaining was performed on 5-μm paraffin-embedded sections after antigen retrieval (boiling in 0.01 M citrate buffer for 10 min). The reactions with primary and secondary fluorescence-labeled antibodies (Supplementary Table 2) followed the protocol described for cultured cells. Immunostaining of outgrowth sections with mouse anti-GFP antibodies was initiated by blocking the sections with the Fab fragment of goat anti-mouse IgG diluted 1:20 (Jackson ImmunoResearch Labs, West Grove, PA) prior to incubation with anti-GFP antibody. After blocking, sections were washed and reacted with horseradish peroxidase-labeled anti-mouse antibody and N-Histofine (Nichirei Biosciences, Tokyo, Japan). Signals were generated with DAB substrate (Vector Laboratories, Burlingame, CA).

For TUNEL assay, paraformaldehyde-fixed cells were permeabilized with 0.2% Triton X-100 (5 min), and apoptotic rhodamine-stained cells were revealed with ApopTag Red *In Situ* Apoptosis Detection Kit (Chemicon, Temecula, CA) according to the manufacturer's protocol.

In all analyses, stained cells were visualized and photographed under an inverted fluorescence microscope (Eclipse Ti, Nikon Instruments, Melville, NY) equipped with NIS-Elements AR 3.2 imaging software (Nikon Instruments), or an Olympus IX 81inverted laser scanning confocal microscope (FLUOVIEW 500, Tokyo, Japan).

### Flow cytometry

Lin^−^ cell suspension was prepared from #4 mammary glands using the EasySep Mouse Mammary Enrichment Kit (StemCell Technologies, Vancouver, Canada) according to the manufacturer's protocol. Antibodies to the mouse hematopoietic cell-surface antigens CD45, CD31 and TER119 enabled elimination of the mouse hematopoietic cells. Lin^−^ mammary cell suspension or CID-9 epithelial cell suspension was resuspended in HF solution (10^7^ cell/ml). Propidium iodide (Sigma) or eFluor 780 (Affymetrix eBioscience, San Diego, CA) staining was performed to mark dead cells, and cell clumps were excluded by filtration through the metal mesh. To characterize the H2AX–GFP-expressing cells within the mammary cell populations and sort them, Lin^−^ cell suspension was labeled with PE-conjugated anti-CD24 and FITC-conjugated anti-CD49f antibodies (Supplementary Table 2). Cell sorting and cell analyses were performed in a FACSAria II cell sorter and LSR II flow cytometer (BD Biosciences), respectively, at the Department of Biological Services of the Weizmann Institute of Science (Rehovot, Israel). Resulting data were visualized and analyzed by FACSDiva (BD Biosciences) and WinMDI 2.9 (Scripps Research Institute, La Jolla, CA) software.

### RNA extraction from mammary cells and real-time PCR analyses

RNA was extracted from mammary gland tissue and cultured cells using the TRIzol reagent and reverse-transcribed with SuperScript II Reverse Transcriptase (Life Technologies, Gibco BRL) as previously described [25]. RNA quality and quantity were determined in a NanoDrop 1000 spectrophotometer (Thermo Fisher Scientific, Wilmington, DE). Quantitative real-time PCR analyses were performed in a StepOnePlus instrument (Applied Biosystems, Foster City, CA) in a 10 ml reaction volume containing 3 μl cDNA (diluted 1:100), 7 μl SYBR Green fast PCR Master Mix (Applied Biosystems) and 10 μl of the primers listed in Supplementary Table 1. The thermal cycling conditions consisted of 20 s at 95°C followed by 40 cycles of 3 s at 95°C and 30 s at 60^°^C. The amplification curves for the selected genes were parallel. EIF4E was used as the endogenous control. Fold change (relative expression) was calculated using StepOne v2.1 and DataAssist v2.0 software (Applied Biosystems).

### DNA methylation analysis

Total DNA was isolated from CID-9 cells using UltraClean Tissue and Cells DNA Isolation Kit (Mo Bio Laboratories, Carlsbad, CA) according to the manufacturer's protocol. The purified DNA (100 ng of each sample) was processed for bisulfite modification using the Imprint DNA Modification Kit (Sigma) according to the manufacturer's instructions.

The DNA methylation status of the H2AX–GFP promoter was evaluated by PCR amplification with the forward primer 5′-GATTTAAATTTTTTAGAAATTGTAGAGGTA-3′ aligning at the H2AX promoter (−376 bp upstream of the first GFP codon) and the reverse primer 5′- ACCAAAATAAACACCACCCC-3′ aligning at the GFP coding region (+51 bp downstream of the first GFP codon). PCR amplification of the 427-bp fragment of the H2AX–GFP promoter was performed using KAPA SYBR FAST Master Mix (Kapabiosystems Inc., Boston, MA) under the following conditions: initial incubation at 95°C for 5 min, 40 cycles of denaturation at 95°C for 20 s, annealing at 58°C for 20 s, and elongation at 64°C for 45 s. The PCR products were separated on an agarose gel (1.5%), extracted from the gel with the QIAquick Gel Extraction Kit (Qiagen, Hilden, Germany) and cloned into PGEMT-easy vectors (Promega, Madison, WI) according to the manufacturer's protocol. PGEMT-easy vectors were multiplied using JM109-competent *Escherichia coli* cells (Promega) and then purified from the bacteria with the QIAprep Spin Miniprep Kit (Qiagen). H2AX–GFP promoter sequence and methylation status of the 25 CpG sites (8 clones from each sample) were analyzed by sequencing (Hylabs, Rehovot, Israel).

### Statistics

Unless otherwise indicated, Student's *t*-test was performed for statistical analyses.

## SUPPLEMENTARY FIGURES AND TABLES


