# Supplementary material for: Luminal STAT5 mediates H2AX promoter activity in distinct population of basal mammary epithelial cells
Source: Oncotarget. 2016 May 30;7(27):41781–97. doi: 10.18632/oncotarget.9718 (PMC5173096; doi:10.18632/oncotarget.9718)
Supplement: Supplementary file 1 [file oncotarget-07-41781-s001.pdf]

Luminal STAT5 mediates H2AX promoter activity in distinct population of basal mammary epithelial cells

Supplementary Materials

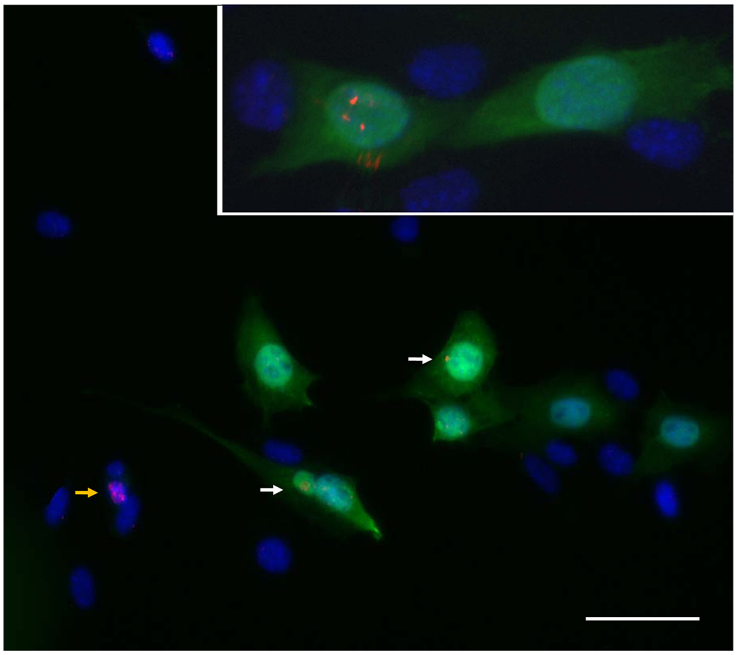

Supplementary Figure S1: TUNEL analysis of H2AX–GFP-enriched BLG–STAT5ca-transfected cultures. GFP$^+$ (white arrow) and GFP$^-$ (yellow arrow) cells with TUNEL-positive nuclei are shown. Inset: 2.5X magnification of TUNEL-positive and negative GFP$^+$ cells. Bar = 50 µm.

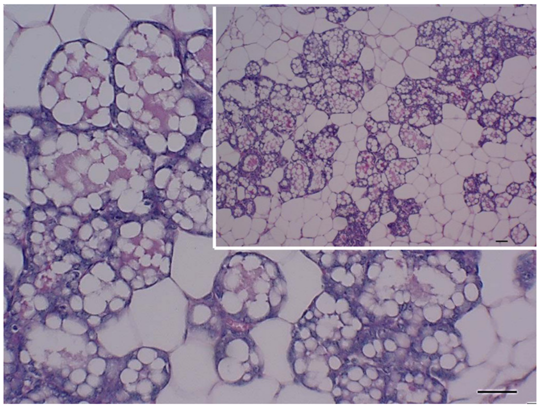

Supplementary Figure S2: Morphology of the pregnant mammary gland. H&E staining of 18-day pregnant mammary gland. Bar = 50 µm. Inset: twofold lower magnification.

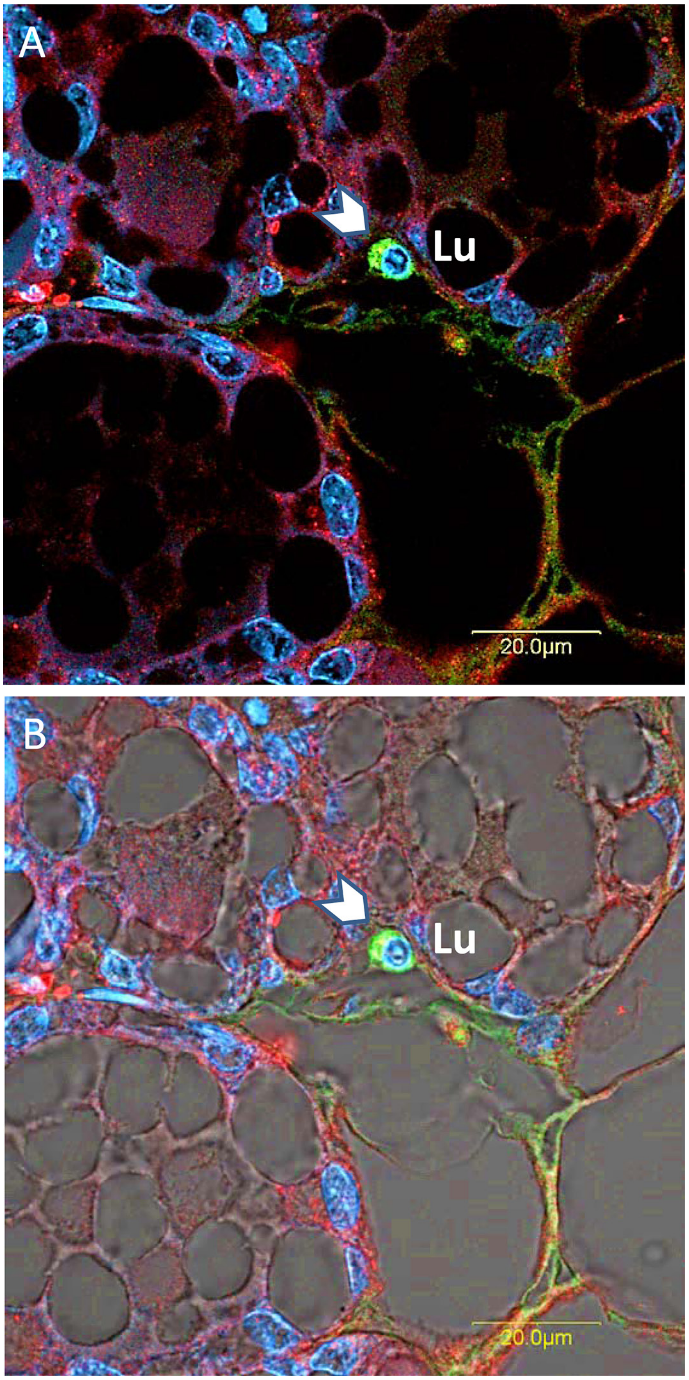

Supplementary Figure S3: H2AX and CK18 are localized in basal and luminal compartments, respectively, in the lobuloalveolar structure of the pregnant mammary gland. Dark field (**A**) and bright field (**B**) images of H2AX-expressing cell (green, marked by arrowhead) located basal to the CK18-stained (red) luminal cells. Lu, lumen.

Supplementary Table S1: Primers used in this study

Gene	Forward primer (5′→3′)	Reverse primer (5′→3′)	Product size (bp)
H2AX promoter (amplification)	TGTTGTGATTGGGAAGCGTAGA	GATCTCGGCAGTGAGGTACTCG	1425
H2AX promoter (restriction site generation)	CCACTGAACCATATGCCAAGTCTGGT	CGCTGAGAGACCACGAGCTAGCAACC	1039
H2AX–GFP detection (transgenics)	CGTCACCGAAGGGTTAATTG	GTGGTGCAGATGAACTTCAGG	304
GFP detection	GTCCGCCCTGAGCAAAGA	TCCAGCAGGACCATGTGATC	74
H2AX promoter	GGCGCGTTCTCTCAGCTAAG	CACCTCTGGTTGGTTGCAATT	58
H2AX coding	CGCAGGCCTCTCAGGAGTA	AGGGCCTTTGTGGAGGTGT	75
CHK2	ACATGAAGAAGAAATTTCAGGATT	CGCTTTTGACTGGAAGTCTG	91
EIF4E	CAGGAGGTTGCTAACCCAGA	TGCTTGCCAAGTTTTGCTT	98
BRCA1	TCCGTGGTGAAGGAGCTT	GCAGTTGCTGTCTTCTGTCC	96
RANKL	CATTTGCTTTCGGCATCATG	TTGGGATTTTGATGCTGGTTT	94

Supplementary Table S2: Antibodies used in this study

Antigen	Primary antibody	Manufacturer	Dilution	Secondary antibody	Manufacturer	Dilution
GFP	Mouse monoclonal, cs-9996	Santa Cruz Biotechnology Santa Cruz, CA	1:100	Cy3-conjugated donkey anti-mouse IgG	Jackson ImmunoResearch West Grove, PA	1:500
GFP	Rabbit monoclonal, ID 2956	Cell Signaling Technology, Beverly, MA	1:100	Alexa Fluor 488-conjugated goat anti-rabbit IgG	Molecular Probes Eugene, OR	1:500
H2AX	Rabbit monoclonal, ID 7631	Cell Signaling	1:100	Alexa Fluor 488-conjugated goat anti-rabbit IgG	Molecular Probes	1:500
CD24	PE-conjugated, rat monoclonal, clone M1/69	StemCell Technologies, Vancouver, Canada	1:50	N/A	N/A	N/A
CD49f	PE-conjugated, rat monoclonal, ID 555736	BD Pharmingen, San Diego, CA	1:50	N/A	N/A	N/A
αSMA<br>CK14	Rabbit Orb163284<br>Mouse anti- human monoclonal	Biorbyt, Cambridge, UK<br>AbD Serotec Kidlington, UK	1:150<br>1:50	Alexa Fluor 488-conjugated goat anti-rabbit IgG	Molecular Probes	1:500
CK18	Chicken polyclonal, ab14047	Abcam, Cambridge, UK	1:130	Alexa Fluor 555-conjugated goat anti-chicken IgG	Molecular Probes	1:1000
P-Stat5	Rabbit monoclonal, ID 9314	Cell Signaling	1:200	Alexa Fluor 488-conjugated goat anti-rabbit IgG	Molecular Probes	1:500

N/A – Not applied.
